# Inclined treadmill walking kinetics of the non-paretic leg in early post-stroke survivors: an observational case-control study

**DOI:** 10.7717/peerj.20766

**Published:** 2026-02-13

**Authors:** Jiani Lu, Yun Miao, Dingying Ma, Lihua Chen, Bo Yu, Jung H. Chien

**Affiliations:** 1 Department of Rehabilitation, Shanghai General Hospital, Shanghai Jiao Tong University School of Medicine, Shanghai, China; 2 Department of Rehabilitation, Ningbo Ninth Hospital, Ningbo, Zhejiang Province, China; 3 Department of Rehabilitation, Shanghai Fifth Rehabilitation Hospital, Shanghai, China; 4 Life University, Marietta, GA, United States of America

**Keywords:** Stroke, Treadmill, Incline, Ground reaction force

## Abstract

**Background:**

Stroke remains the primary source of prolonged disability worldwide since it often results in gait asymmetry. Research shows compensatory mechanisms in chronic stroke patients’ non-paretic legs but lacks knowledge about these adaptations during the early post-stroke period when walking on inclines which mimic real-world mobility challenges.

**Objective:**

This study sought to clarify the compensatory strategies in the non-paretic leg of early post-stroke survivors by analyzing the changes in vertical ground reaction force (GRF) profiles and respective variabilities during level (0%) and inclined (6%) treadmill walking, relative to healthy, matched controls.

**Methods:**

The study included fourteen early post-stroke survivors who were three months post-event or less along with fourteen matched controls. Participants walked at their preferred speeds on treadmill at 0% and 6% grade incline settings. Researchers determined key vertical GRF profiles, including peak amplitudes (F1, F2, F3), impulses (J1–J4), timing to each peak, loading/unloading rates, and their respectively variabilities. Mixed two-way repeated measures Analysis of covariance (ANCOVA) were used and preferred walking speed was used as a covariate.

**Results:**

Stroke survivors demonstrated significantly slower walking speeds compared to control participants (0.6 km/hr *vs.* 2.15 km/hr) while both groups shared similar demographic characteristics. It is worth mentioning that this study specifically focused on the non-paretic side; therefore, only GRF components corresponding to the non-paretic leg of the stroke survivors were investigated. During weight acceptance (F1) and push-off (F3) phases, stroke survivors showed lower vertical ground reaction force (VGRF) peak amplitudes (F1- control (C): 1.11 *vs.* patients (P): 1.04, F3 - C: 1.09 *vs.* P: 1.03) and impulse magnitudes (J1- C: 25.46 *vs.* P: 18.74, J2 - C: 21.37 *vs.* P: 13.46, J3- C: 22.38 *vs.* P: 15.71, J4 - C: 22.16 *vs.* P: 18.19) in their non-paretic leg compared to control subjects regardless of inclines. Stroke survivors presented higher F2 (mid-stance, F2 - C: 0.92 *vs.* P: 0.98) values which might indicate a flatter vertical ground reaction force profile due to compensatory or pathological gait mechanisms. Inclined walking produced increased F1 (Grade 0%: 1.06 *vs.* Grade 6%: 1.10) and decreased F2 (Grade 0%: 0.97 *vs.* Grade 6%: 0.93) for both participant groups, but controls exhibited increased F3 (Grade 0%: 1.06 *vs.* Grade 6%: 1.12) while stroke survivors showed reductions in F3 (Grade 0%: 1.05 *vs.* Grade 6%: 1.01) which indicated impaired push-off mechanics.

**Conclusions:**

The study demonstrates that treating the non-paretic leg as a healthy limb is inadequate and shows the necessity for rehabilitation approaches that target both legs individually. Focusing on motor control and force steadiness instead of simply strength can more effectively reduce maladaptive variability and enhance safe and efficient walking patterns.

## Introduction

In the United States, stroke still presents a major public health concern. About 7.09 million Americans were suffering from stroke-related effects as of 2019 (Renedo et al., 2024). About 795,000 Americans have strokes each year. Demographic changes toward an older population and the ongoing presence of risk factors like obesity, diabetes, and hypertension are expected to drive increases in this number in the following decades. Over fifty percent of survivors aged 65 and above have limited mobility, so stroke is the main cause of major long-term disability recorded globally (Cho et al., 2024). One of the most common and functionally limiting effects of stroke is gait asymmetry, which often develops as a compensatory mechanism. Of those who have chronic stroke, over half show asymmetries in trunk movement, stance duration, and stride length (Cho et al., 2024). These deviations from symmetrical gait patterns usually cause discomfort and later musculoskeletal problems, like impairing balance, increasing the metabolic cost of ambulation, and exerting great mechanical stress on the non-paretic leg (Ju, 2020).

From a biomechanical standpoint, stroke patients clearly exhibit compensating mechanisms in both kinematic and spatiotemporal parameters due to the asymmetric gait. Kinematic studies show notable differences in joint angles, movement trajectories, and interlimb variability across the gait cycle in chronic stroke survivors (Kim & Eng, 2004; Cho et al., 2024). By spatiotemporal parameters, frequent observations of reduced step length and single-limb stance duration on the paretic side, together with a compensatory increase in double support time to improve stability in the non-paretic leg, clarify the type of post-stroke gait asymmetry (Alexander et al., 2009; Crosby et al., 2020; Zhu et al., 2025). These abnormalities point to ongoing temporal gait asymmetry, which directly relates to poor energy use, and the development of secondary musculoskeletal problems. Furthermore, tightly linked to the degree of motor and balance deficits, these asymmetries usually remain even with traditional rehabilitation programs are applied (Alexander et al., 2009; Crosby et al., 2020; Zhu et al., 2025). Comprehensive recording of kinematic and spatiotemporal data is made possible by advanced motion capture methods. These conventional marker-based systems, which call for multiple cameras, specialized laboratory space, and trained technical workers, however, have high infrastructure needs, operational complexity, and high expenses. Ground reaction force (GRF) devices have advantages of resource-limited clinical environments because of their practical and reasonably cost approaches for gait assessment.

Different biomechanical patterns identified in GRFs among chronic stroke patients indicate neurological deficits and compensatory responses. Characterized by changed force profiles marked by spatiotemporal asymmetries, which include both decreasing braking and propulsive impulses in both limbs relative to healthy controls, particularly, the non-paretic limb often generates lower GRFs than the dominant limb of healthy individuals due to the slower walking speed (Lee, Chang & Jeon, 2020; Ohta et al., 2023). The results highlight the clinical relevance of GRF analysis in devising biomechanically informed treatments addressing main deficits and secondary compensatory adaptations in post-stroke gait rehabilitation. Specifically, in stroke patients in early stage (Chen et al., 2007), abnormal GRF patterns, such as inverted “V” or “U”-shaped vertical force curves, often replace the normal bimodal (“M”-shaped) distribution in healthy controls. This observation causes the force profiles between non-paretic and paretic leg incomparable in early state of stroke survivors. Therefore, many important limitations still need to be addressed even with the insightful analysis of past studies.

The novelty of this present study lies in studying gait mechanics during the subacute phase (typically within 3 months) of stroke survivors, a period fundamentally different from the stable chronic phase (5 to 8 months and beyond). During the subacute stage, the brain is in a state of maximal neuroplasticity and spontaneous recovery, which results in rapidly improving but highly variable gait parameters (Hendricks et al., 2002; Kwakkel, Kollen & Lindeman, 2004). This period is marked by the formation of new, often unrefined, motor strategies, a pattern that is volatile and highly susceptible to therapeutic intervention (Liu et al., 2023; Wade, Wood & Hewer, 1985). Conversely, at 5 to 8 months, the rate of recovery has significantly plateaued, and the patient’s walking is defined by fixed, ingrained compensatory patterns (Patterson et al., 2015). The studies by Zhang et al. (2022) and Xin et al. (2023) were two pioneering efforts that established the foundational evidence for using inclined walking in this early recovery window. Specifically, Zhang et al. (2022) demonstrated that while patients exhibited severely impaired gait patterns compared to controls, increasing the degree of inclination enhanced their performance, leading to better spatial and temporal characteristics, and significantly reduced step length asymmetry. Additionally, Xin et al. (2023) found that increasing the incline increased peak hip flexion in the paretic leg, a strategy likely used to generate propulsive power; however, they also observed a trade-off, with peak knee flexion in the paretic leg significantly dropping at the 6° incline. This present study builds directly upon those kinematic findings to specifically and comprehensively explore the GRFs and their profiles in early-stage stroke survivors while walking on different inclines. By investigating GRFs during this subacute phase, the biological mechanisms of active motor recovery and adaptation before compensatory patterns become permanent can be captured. Analyzing the non-paretic limb’s GRFs, which tend to be over-relied upon for stability and propulsion, offers an alternative, clearer way to understand how early intensive training might successfully influence these developing GRF profiles, thereby strengthening the rationale for incline training in early stroke rehabilitation.

This work sought to clarify compensating processes by means of vertical GRF changes in the non-paretic leg of early stroke survivors relative to age-, weight-, and height-matched controls. The study looked at how early stroke patients modified their compensatory techniques on a gradient usually found on pedestrian paths, a 6% incline. The theory put up was that independent of walking on level ground or a 6% slope, stroke survivors would have smaller GRF peak and impulse amplitude than controls. Moreover, it was expected that time to these peaks would be delayed in the weight acceptance (braking) phase and pushing off (accelerating) phase. Stroke survivors were supposed to have more GRF variability than controls; moreover, a decrease in GRF variability was expected with increasing inclination.

## Materials & Methods

### Participants

Fourteen stroke survivors who had experienced a single stroke within the past three months (12 males, two females), along with fourteen age-, weight-, and height-matched controls (12 males, two females), participated in this study. The walking speed was 0.6 km/hr (0.17 m/s) for stroke survivors and 2.15 km/hr (0.6 m/s). The walking speed in these stroke survivors was similar to the speed in a previous study (Conesa et al., 2012). Further participant details are presented in Table 1. The study protocol received approval from the Ethics Committee of Ningbo Ninth Hospital (# 2023LIW13). All participants signed the inform consents. Inclusion criteria were as follows: (1) healthy controls were required to be free from musculoskeletal and cognitive impairments, with no history of extremity injuries or falls within the past year; (2) stroke survivors were required to ambulate independently for at least 10 m and achieve a Functional Independence Measure Locomotor Item score of 5 or higher (Phan et al., 2013; Ada, Dean & Lindley, 2013); and (3) stroke survivors were required to present with gait deficits, defined as walking speeds ≤ 0.8 m/s, a threshold previously associated with community ambulation with asymmetric gait (Perry et al., 1995). Participants were recruited from the inpatient and outpatient rehabilitation clinics of Ningbo Ninth Hospital between February 2023 and January 2024. The matched controls were recruited through the hospital’s newsletter and website. In this study, the cases were the stroke survivors who met the specific inclusion criteria (*e.g.*, within 3 months post-stroke, walking speed ≤0.8 m/s). Since recruiting clinical populations like subacute stroke patients can be difficult and time-intensive, it was logistically efficient to secure the pathological group first. The “matched controls” were selected to be similar to the cases based on criteria believed to confound the results: age (matched within five years), sex (identical between groups), height (matched within five centimeters), and weight (matched within five kilograms). All enrolled stroke patients commenced conventional physical therapy interventions during the acute phase of recovery. The rehabilitation program included range of motion exercises, strengthening and stretching exercises, gait training, transfer training, and activities of daily living (ADL) training.

**Table 1:** Participants’ information: means (standard deviations), the walking speed was used as a covariate.

**Strokes**						Controls				
Day from stroke to data collections (days)	Gender (M: male; F: female)	Age (Years)	Walking speed (km/hr)	Height (cm)	Weight (kg)	Gender (M: male; F: female)	Age (years)	Walking speed (km/hr)	Height (cm)	Weight (kg)
49.43 (18.45)	2F/12M	66.86 (11.29)	0.6 (0.19)	171.5 (6.69)	73.14 (9.95)	2F/12M	66 (11.49)	2.15 (0.8)	171.07 (6.23)	73.64 (9.87)

### Sample size selection

The sample size in the present study was selected based on previous studies (Faria et al., 2016; Chow & Stokic, 2015). Both previous studies recruited fourteen stroke survivors compared to controls. In Faria et al.’s study (2016), a two-way mixed ANOVA was used to compare the groups (stroke *vs.* control) and turning directions (different locomotor tasks). Similarly, Chow & Stokic (2015) applied a one-way ANOVA to investigate the main effect of groups (paretic, non-paretic, and control) for spatial–temporal gait parameters, joint ranges of motion, and ranges of peak timing of elevation angles while participants were instructed to walk with elevation angles of the thigh, shank, and foot segments in the sagittal plane. Both studies found a significant difference in gait parameters between stroke survivors and controls. Importantly, the *p*-values were approximately 0.001, suggesting a large effect size. Therefore, recruiting 14 subacute stroke survivors and 14 controls in the present study should be sufficiently powered to compare the between-group results.

### Data collection

**Figure 1:**
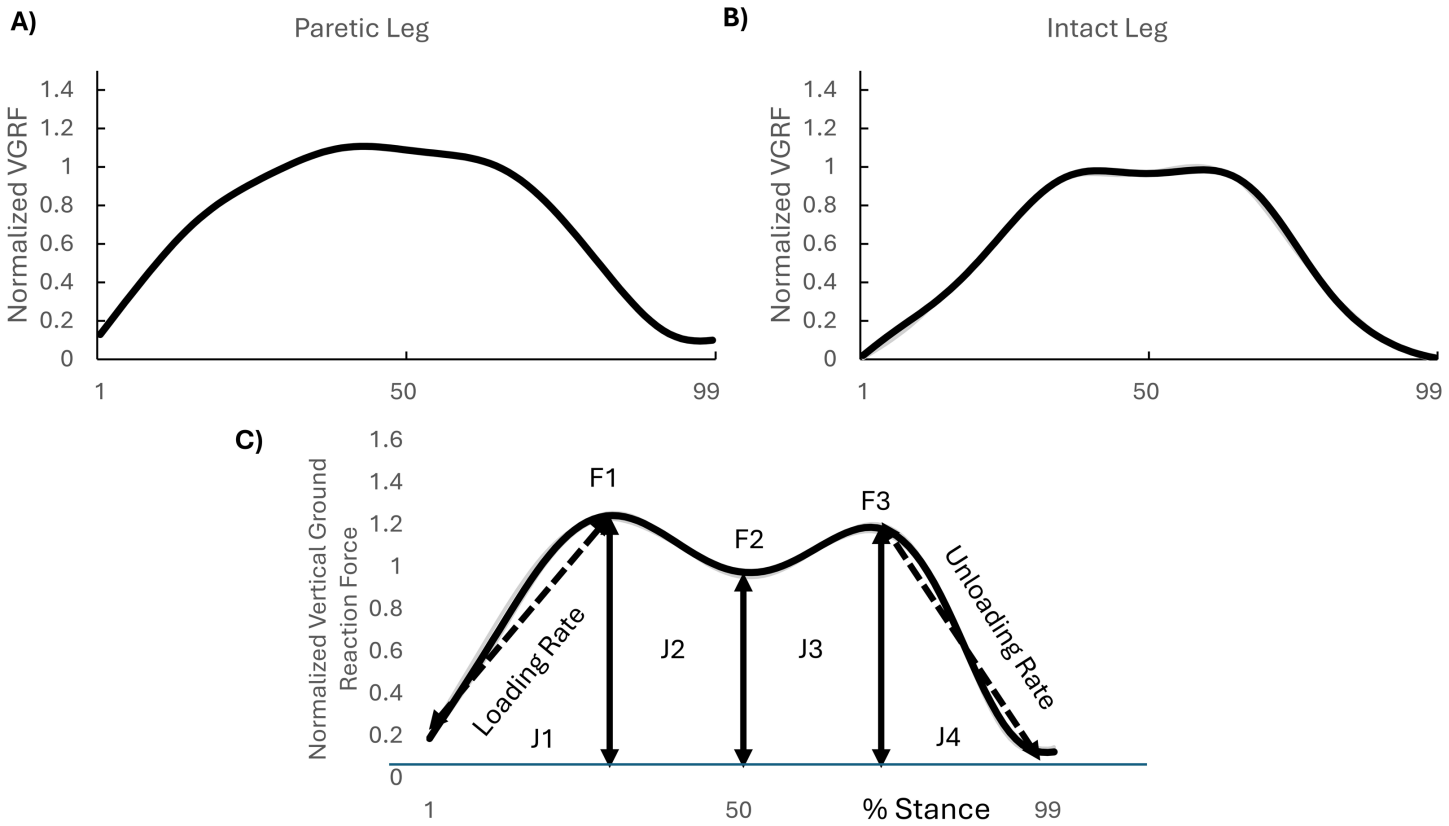
Different ground reaction force profiles. Vertical ground reaction force in paretic leg (A) and in the intact leg (B). (C) the interest points in dominant leg in healthy young and non-paretic leg in stroke survivors in early stage. J1–J4 represented the area under curves.

Vertical ground reaction force (VGRF) was measured using a Zebris FDM-T treadmill (Zebris Medical GmbH, Germany), a system previously validated for the assessment of force-related parameters and center of pressure in various clinical populations, including individuals with multiple sclerosis (Kalron et al., 2013) and with Down syndrome (Wu & Ajisafe, 2014). The treadmill is equipped with a 150  × 50 cm running surface, allowing for adjustable speeds from 0.2 to 22 km/h in 0.1 km/h increments, and supports inclines ranging from 0% to 20%. Its pressure-sensing platform, also 150  × 50 cm, contains 10,240 individually calibrated sensors, enabling high-resolution mapping of plantar pressure. Pressure data were sampled at 120 Hz and processed in real time using the RehaWalk-Gait Analysis software (Zebris Medical GmbH, Germany; see Fig. 1), facilitating the detection of subtle changes in force distribution across the foot (Kalron et al., 2013; Wu & Ajisafe, 2014).

Preferred overground walking speeds were assessed for both healthy controls and stroke patients using a pressure-sensing walkway system, an electronic walkway (GAITrite, CIR System Inc., Clifton, NJ, USA), containing a total of 13,824 sensors sensor pads with an active sensor area of 366 cm long and 61 cm wide, is connected to the USB port of computer. As participants walked along the walkway, plantar pressure distributions were recorded at 80 Hz and transmitted to a computer for analysis. After providing informed consent, participants completed five trials of a 10-meter walk test along a straight path marked by tape between two points spaced 10 m apart. This procedure was designed to evaluate preferred walking speeds (overground PWS) and step lengths using the pressure sensor-embedded walkway.

Following the initial assessment of the overground PWS, a PWS for the treadmill needed to be set up. First, participants stood on the side of the treadmill. Then, the treadmill was inclined to a 6% grade and experimenters increased the treadmill speed from 0 to the overground PWS. Participants held onto the handrail and stepped onto the treadmill belt. After 20 s, experimenters asked whether the speed they were experiencing was comfortable, such as walking around their backyard with inclines. Based on the participants’ responses, experimenters increased or decreased the speed by 0.1 km/hr. The procedure was repeated until the participants confirmed the PWS. Once the PWS was identified, participants took a five-minute mandatory sitting rest. Next, participants who met the inclusion criteria needed to complete two locomotor tasks: walking on 0% and 6% grade inclines. To mitigate potential order effects, the walking tasks (level *vs.* 6% incline) were systematically counterbalanced, ensuring half of the participants completed the level condition first and the other half completed the incline condition first. Each trial lasted two minutes suggested by Kempski et al.’s study (2018) in patients with stroke. Randomization procedures were applied equally to both healthy controls and stroke participants. There was a two-minute mandatory rest for catching up the breath between experimental trials for all participants. An extended rest would be provided if participants required it. Importantly, participants were encouraged to refrain from holding the handrail during the assessment. To ensure safety, two physical therapists were positioned alongside each stroke survivor, with one therapist responsible for operating the emergency stop mechanism. Participants were informed that they could halt data collection at any time in the event of discomfort or respiratory distress, especially while walking on the 6% graded incline.

### Data analysis

**Figure 2:**
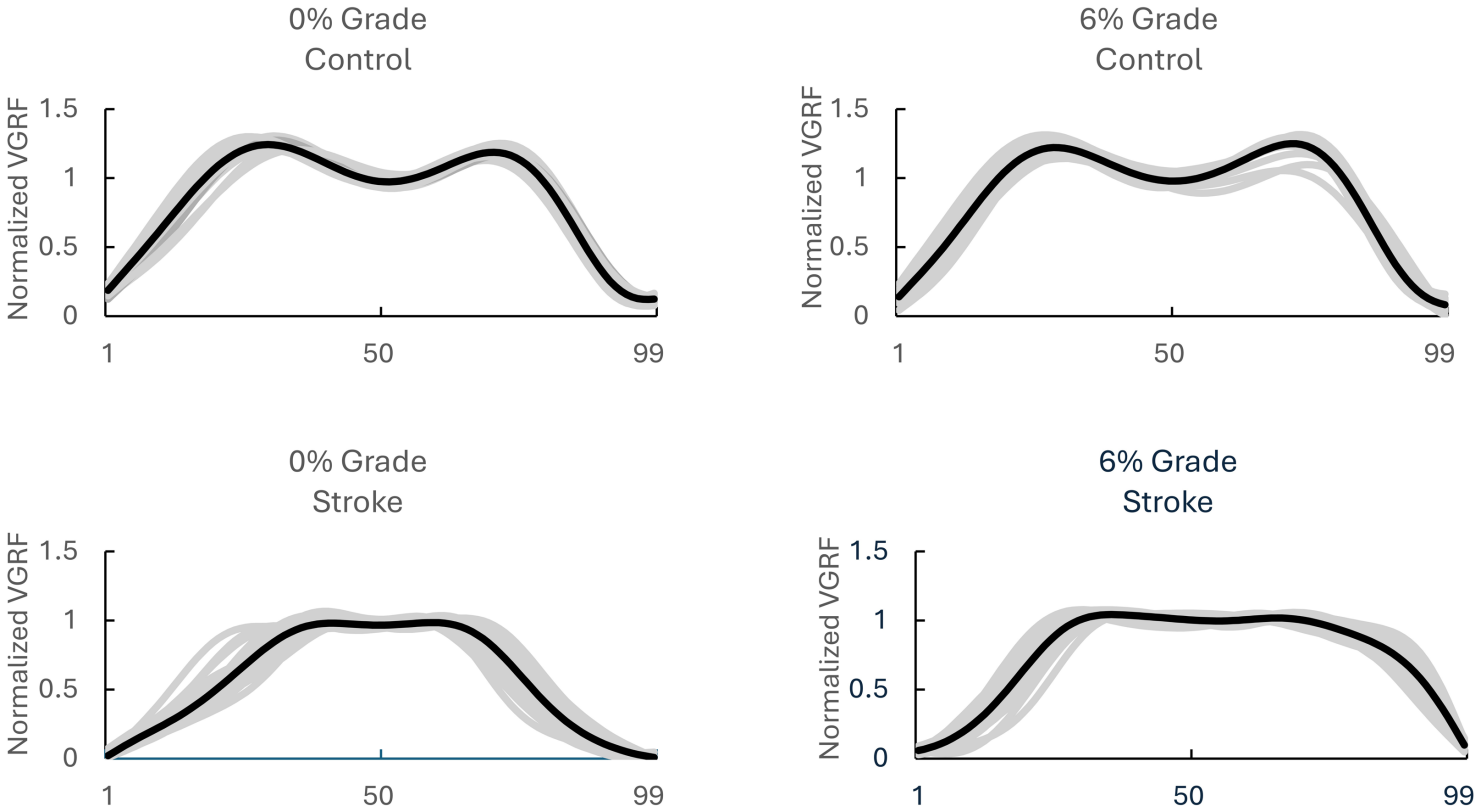
Different ground reaction force profiles between different groups and different incline. Four illustrations representing the vertical ground reaction force when one stroke survivor or one healthy control walked on 0% grade or 6% grade of incline.

It is worth mentioning that this study specifically focused on the non-paretic side; therefore, only GRF components corresponding to the non-paretic leg of the stroke survivors were investigated. Vertical GRF data were acquired using Zebris FDM-T software and exported into text file format for subsequent analysis. The raw signals underwent filtering *via* a fourth-order, zero-lag Butterworth low-pass filter with a cutoff frequency of eight Hz, followed by temporal normalization to 0–100% of the stance phase duration within the gait cycle (Fig. 1B). A custom MATLAB script (MathWorks, Inc., Natick, MA, USA) was developed to automate data processing and gait event detection. Following established methodology by Chien et al. (2014), heel strike initiation was identified as the time point when the vertical GRF component exceeded 10 N and maintained this threshold continuously for 40 ms. Conversely, toe-off events were defined as the instant when GRF values dropped below 10 N and remained subthreshold for a minimum of 40 ms. All force measurements were normalized relative to individual body weight (BW) to account for interparticipant variability. This standardization facilitated cross-participant comparative analysis of kinetic parameters. A custom MATLAB algorithm (MathWorks, Inc., Natick, MA, USA) was employed to identify the amplitude and temporal occurrence (% stance phase) of vertical ground reaction force peaks (F1, F2, F3), along with their respective time-to-peak intervals (time to F1, time to F2, time F3), using methodologies adapted from Wu & Ajisafe (2014) (Fig. 1C). Importantly, it has been shown that stroke survivors presented higher F2 (mid-stance) vertical GRF values, indicating a flatter vertical ground reaction force profile, primarily in the paretic leg (Lauzière et al., 2014) (Fig. 2). This raised a crucial question: was the typical ‘M’-shaped profile with distinct F1, F2 (valley), and F3 peaks consistently identifiable in all participants, especially the stroke survivors? This study utilized three distinct conditions to classify the ‘M’-shape profile: (1) F1 (First Peak): A local maximum must be identified in the first half of the stance phase (0%–50%) using the max function in MATLAB. For all inclination conditions (0% and 6% inclines), this peak must be located between 20% and 45% of the stance phase, (2) F3 (Second Peak): A local maximum must be identified in the second half of the stance phase (50%–100%). For all inclination conditions, this peak must be located between 55% and 80% of the stance phase, (3) F2 (Valley): A local minimum must be identified using the min function in MATLAB between the F1 and F3 peaks. The F2 value was required to be strictly less than both F1 and F3, without any tolerance threshold. For instance, in one gait cycle for stroke survivor #14, the F1 value was 1.068, the F2 value was 1.041, and the F3 value was 1.047. If any of these conditions were violated, the GRF shape was recorded as ‘not-M-shape shape.’

The instantaneous loading rate was calculated as the slope between heel strike and F1, while the unloading rate represented the slope between F3 and toe-off. GRF impulses were partitioned into four distinct phases (Fig. 1C):

J1: Heel-strike to F1

J2: F1 to F2

J3: F2 to F3

J4: F3 to toe-off

Impulse magnitudes were computed *via* numerical integration of force-time curves across specified intervals using MATLAB’s trapz function, which implements the trapezoidal method for discrete dataset integration. One stroke survivor was able to complete only 60 gait cycles while walking on the 6% incline. Consequently, for consistency, analyses were based on 60 stance phases from the non-paretic leg of stroke survivors and the dominant leg of healthy controls even they completed 2 min walking. The amount of GRF variability was quantified as the coefficient of variation calculated across these 60 stance phases. The dominant leg was defined as the leg preferred for kicking a soccer ball.

### Statistical analysis

The Shapiro–Wilk was used to test the normal distribution. If the data was normally distributed, a mixed two-way repeated Analysis of covariance (ANCOVA) measure was used to calculate the interaction between the effect of inclines and the effect of stroke on GRF profiles and respective variabilities. The walking speed was used as a covariate. The post hoc comparisons were corrected by Bonferroni method. Otherwise, a Friedman’s test would be used. If the interaction was significant, the pairwise comparisons using Tukey method were used for normal distributed data or using Mann–Whitney test for the between groups and using Wilcoxon test for the within different inclines for non-parametric data. The alpha level was set 0.05. An effect size was estimated using partial eta squared method: *η*^2^ = 0.01 indicated a small effect, *η*^2^ = 0.06 indicated a medium effect, and *η*^2^ = 0.14 indicated a large effect size.

## Results

The F1–F2–F3 pattern (M-shape pattern) of the non-paretic leg was consistently identifiable in all participants, including stroke survivors, defined by an F2 amplitude that always remained lower than both F1 and F3 across all trials. Thus, no ‘not-M-shape shape’ was reported from either group in this study.

### Normality test

The results of Shapiro–Wilk showed that the normal distribution for all dependent variables (*p* > 0.05, Table 2).

**Table 2:** The *p* values for Normality Test. XV: variability of X, X can be F1, F2, F3, TimetoF1, TimetoF2, TimetoF3, Loading, and Unloading, J1, J2, J3, and J4.

	F1	F1V	F2	F2V	F3	F3V		
Cgrade0	0.46	0.96	0.08	0.53	0.16	0.34		
Cgrade6	0.69	0.06	0.16	0.92	0.61	0.74		
Pgrade0	0.77	0.71	0.06	0.74	0.11	0.09		
Pgrade6	0.34	0.14	0.89	0.42	0.72	0.34		
	TimeToF1	TimeToF1V	TimeToF2	TimeToF2V	TimeToF3	TimeToF3V		
Cgrade0	0.9	0.1	0.21	0.09	0.11	0.46		
Cgrade6	0.33	0.47	0.53	0.87	0.59	0.44		
Pgrade0	0.83	0.25	0.07	0.38	0.99	0.12		
Pgrade6	0.51	0.23	0.34	0.12	0.9	0.99		
	Loading	LoadingV	Unloading	UnloadingV				
Cgrade0	0.21	0.53	0.73	0.44				
Cgrade6	0.53	0.14	0.88	0.76				
Pgrade0	0.68	0.06	0.91	0.96				
Pgrade6	0.12	0.16	0.4	0.72				
	J1	J1V	J2	J2V	J3	J3V	J4	J4V
Cgrade0	0.49	0.13	0.57	0.09	0.73	0.07	0.14	0.16
Cgrade6	0.15	0.59	0.29	0.71	0.68	0.44	0.15	0.16
Pgrade0	0.33	0.17	0.13	0.18	0.61	0.43	0.84	0.73
Pgrade6	0.64	0.33	0.56	0.44	0.25	0.08	0.83	0.27

### Case-control analysis

**Figure 3:**
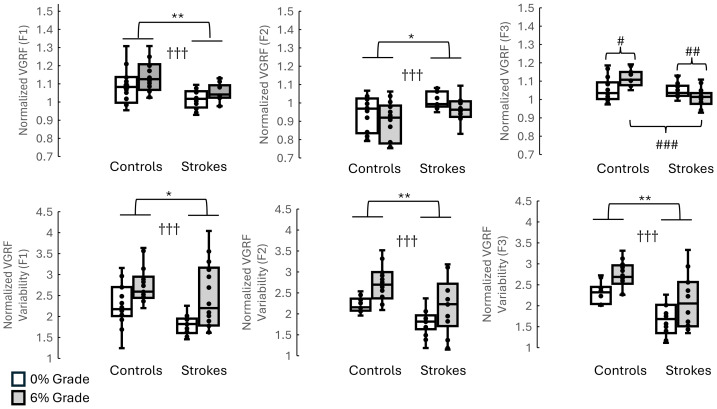
F1, F2, F3 and respective variability between groups and inclines (without using walking speed as a covaraite). The top three graphs illustrate the F1, F2, and F3 between stroke survivors and healthy controls when walking on 0% and 6%. The bottom three graphs represent the F1, F2, F3 variability between stroke survivors and healthy controls when walking on 0% and 6%. Indicates the significant differences between different inclines. * indicates the significant difference between groups. *: *p* < 0.05, **: *p* < 0.01, ***: *p* < 0.001. # indicates the pairwise comparisons after the interaction was significant. #: *p* < 0.05, ##: *p* < 0.01, ###: *p* < 0.001.

**Figure 4:**
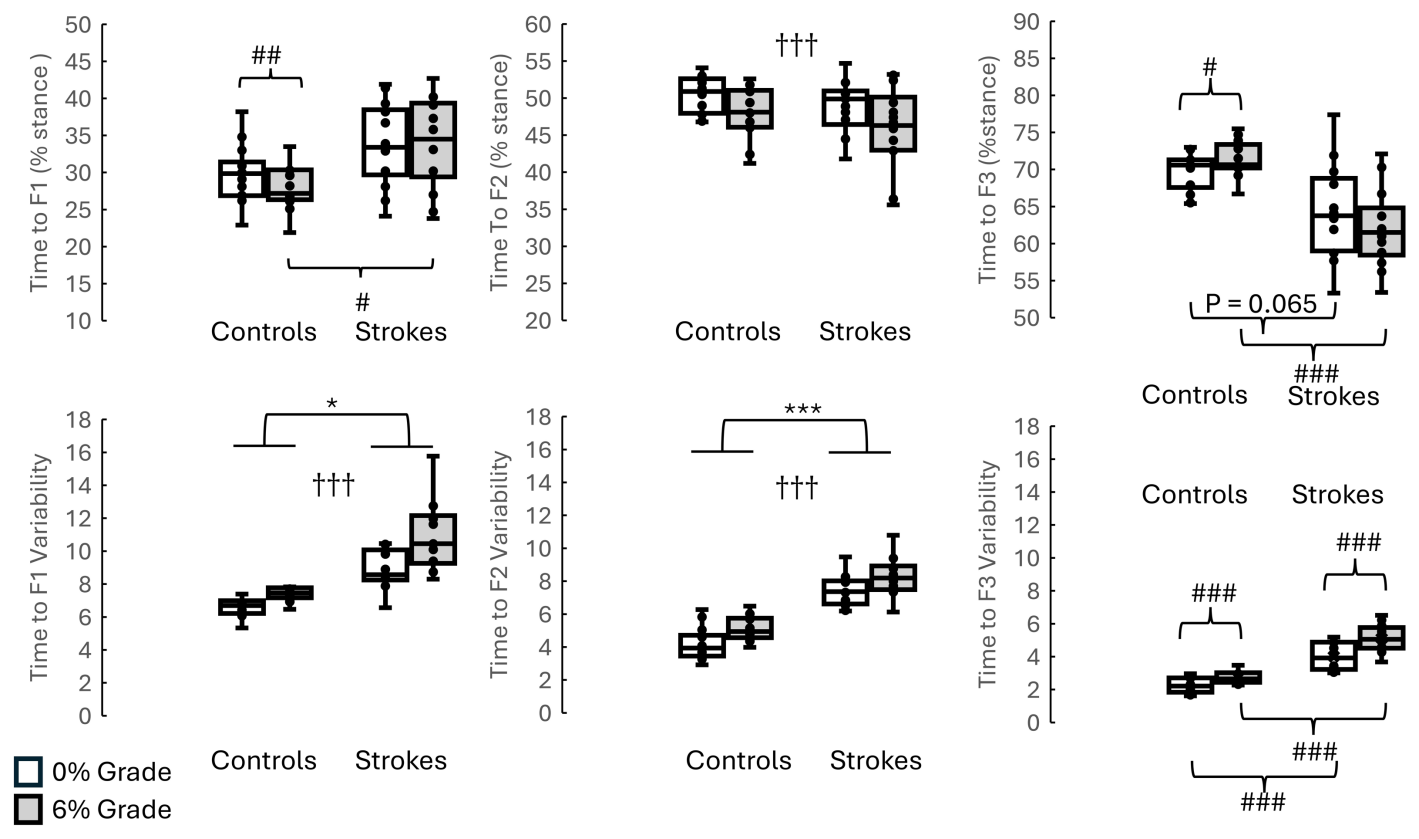
Time to F1, Time to F2, Time to F3 and respective variability between groups and inclines (without using walking speed as a covaraite). The top three graphs illustrate the Time to F1, Time to F2, and Time to F3 between stroke survivors and healthy controls when walking on 0% and 6%. The bottom three graphs represent the Time to F1, Time to F2, Time to F3 variabilities between stroke survivors and healthy controls when walking on 0% and 6%. Indicates the significant differences between different inclines. * indicates the significant difference between groups. *: *p* < 0.05, **: *p* < 0.01, ***: *p* < 0.001. # indicates the pairwise comparisons after the interaction was significant. #: *p* < 0.05, ##: *p* < 0.01, ###: *p* < 0.001.

**Figure 5:**
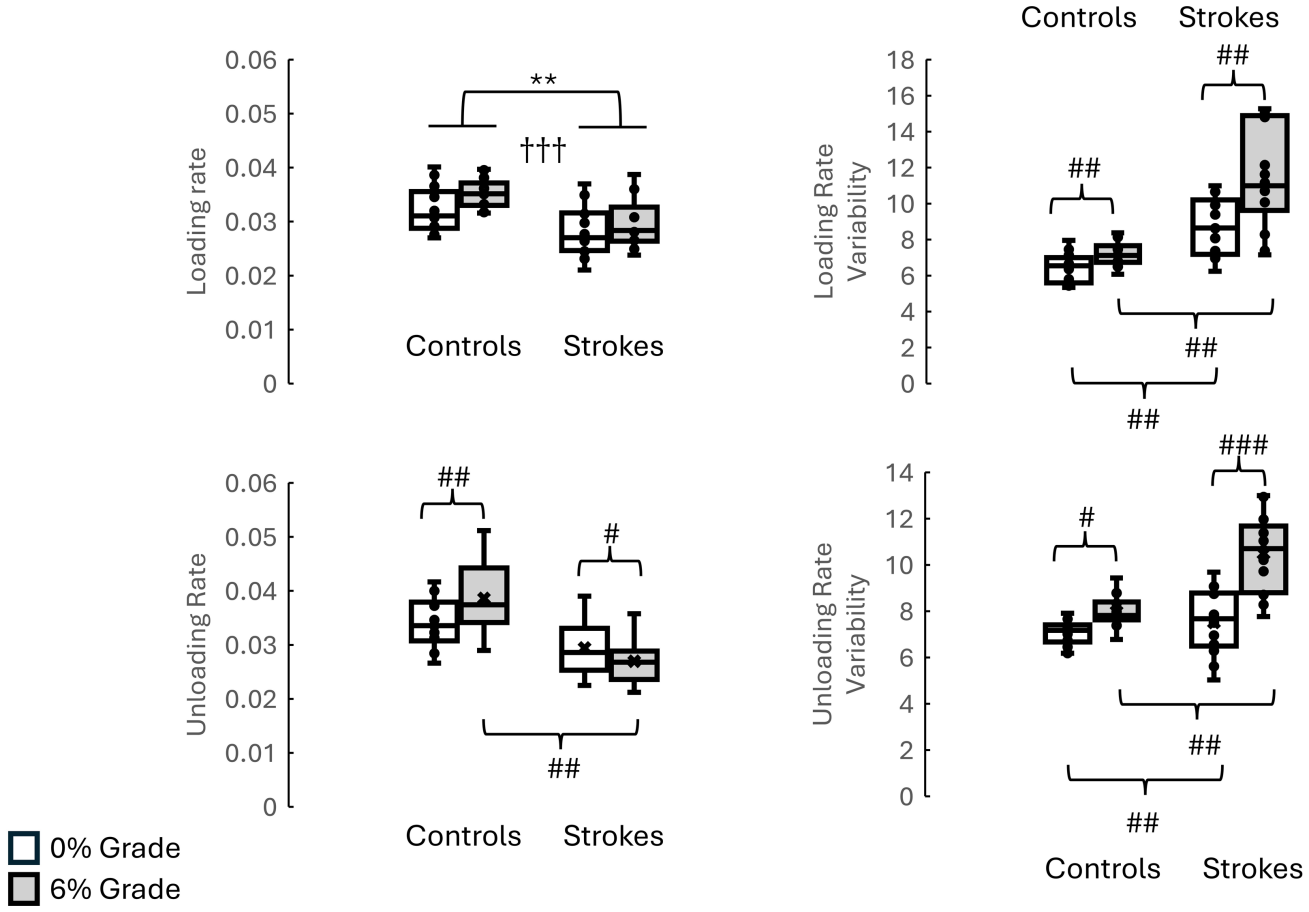
Loading and unloading rates and respective variability between groups and inclines (without using walking speed as a covaraite). The top two graphs illustrate the loading rate and unloading rate between stroke survivors and healthy controls when walking on 0% and 6%. The bottom two graphs represent the loading rate and unloading rate variabilities between stroke survivors and healthy controls when walking on 0% and 6%. Indicates the significant differences between different inclines. * indicates the significant difference between groups. *: *p* < 0.05, **: *p* < 0.01, ***: *p* < 0.001. # indicates the pairwise comparisons after the interaction was significant. #: *p* < 0.05, ##: *p* < 0.01, ###: *p* < 0.001.

The results showed that (1) smaller means in stroke survivors than in controls were found in F1(*p* = 0.035), F2 variability (*p* = 0.025), F3 variability (*p* = 0.019), time to F3 (*p* = 0.014), J1 (*p* = 0.037), J2 (*p* = 0.011), J3 (*p* = 0.038), and J4 (*p* = 0.001); and (2) greater means in stroke survivors than in controls were observed in F2 (*p* < 0.001), time to F1 (*p* = 0.007), time to F1 variability (*p* < 0.001), time to F2 variability (*p* < 0.001), time to F3 variability (*p* < 0.001), J1 variability (*p* = 0.032), J2 variability (*p* < 0.001), J3 variability (*p* = 0.004), and J4 variability (*p* < 0.001). The effect of walking speed as covariate was found in F1 (*p* < 0.001), F2 (*p* = 0.001), and J1 (*p* < 0.001). More detailed results are shown in Figs. 3–5 and Table 3.

### The interaction between groups (stroke *vs.* controls) and conditions (level *vs.* 6 grade incline)

The detailed results of the interaction between groups and conditions are shown in Figs. 3–5 and Table 3. The pairwise comparisons results showed that (1) as the surface incline increased, F3 increased in controls but decreased in stroke survivors, (2) as the surface incline increased, the time to F3 increased in controls but decreased in stroke survivors, and (3) when walking on a 6% incline, stroke survivors demonstrated the lowest unloading rate but the highest unloading rate variability. The *η*^2^ for interactions was 0.273 for F3, 0.172 for Time to F1, 0.366 for Time to F3, 0.599 for unloading rate, 0.303 for J3, and 0.803 for J4. These *η*^2^ indicated the large effect in this present study.

### The effect of inclines

All results were presented in Figs. 3, 4 and 5 and Table 3. The pairwise comparisons showed that walking on a 6% grade increased the following variables more than walking on a 0% incline: F1 (*p* = 0.044), F1 variability (*p* = 0.007), F2 (*p* = 0.006), F2 variability (*p* = 0.013), F3 variability (*p* = 0.021), time to F2 (*p* = 0.013), time to F2 variability (*p* = 0.044), time to F3 variability (*p* = 0.018), J1 (*p* = 0.05), and J2 (*p* = 0.003).

### The effect of speed as covariate

A significant effect of speed as a covariate was found in F1 (*p* < 0.001), F2 (*p* < 0.001) and J1 (*p* < 0.001), indicating that a large portion of the observed variance in initial loading force and mid-stance vertical force was linearly related to walking speed. More details are shown in Table 3 and Fig. 3.

## Discussion

Due to the limited information regarding how early stroke survivors adjust their non-paretic leg to compensate for abnormal gait, particularly when walking on different graded inclines, this study aimed to address these gaps in knowledge. The results were largely consistent with the hypotheses that (1) the amplitudes of GRF peaks and impulses were smaller in stroke survivors than in controls, and (2) the time to these peaks would be delayed during the weight acceptance (braking) phase but be shorten during pushing off (accelerating) phase. Unexpectedly, both greater and smaller variabilities were observed across different dependent variables. It was worth mentioning that the analysis utilized a mixed two-way repeated measures ANCOVA to account for the substantial difference in walking speed between the stroke survivors (0.6 km/hr) and healthy controls (2.15 km/hr). Walking speed was found to be a significant covariate for the vertical GRF parameters: F1 (weight acceptance peak, *p* < 0.001), F2 (mid-stance valley amplitude, *p* = 0.001), and J1 (impulse during initial loading, *p* < 0.001). This significance confirmed that a large portion of the observed variance in initial loading force and mid-stance vertical force was linearly related to walking speed. By statistically adjusting for walking speed, the ANCOVA successfully isolated the differences between the stroke and control groups that are genuinely attributable to the pathological and compensatory gait mechanisms following the stroke, providing a cleaner assessment of the effect of the neurological impairment independent of the confounding variable of walking velocity.

### The non-paretic leg in stroke survivors was not equivalent to the dominant leg in healthy individuals

Present investigation revealed higher impulse magnitudes and peak amplitudes in healthy controls than in stroke survivors. These results showed partial concordance with earlier studies by Lee, Chang & Jeon (2020), which investigated GRFs in healthy individuals and chronic stroke survivors (≥7 months post-stroke) during overground walking. Apart from prolonged mid-stance impulse durations, the non-paretic limb revealed reduced vertical GRFs during weight acceptance (F1: 0.96 BW *vs.* 1.09 BW in controls, the difference was 0.13 BW) and push-off phases (F3: 0.96 BW *vs.* 1.11 BW in controls, the difference was 0.15 BW), indicating changed force distribution between stroke survivor and controls (Lee, Chang & Jeon, 2020). By contrast, the present study found decreased between-group differences (F1: 1.04 BW in stroke survivors *vs.* 1.11 BW in controls, the difference was 0.07 BW; F3: 1.03 BW in stroke survivors *vs.* 1.09 BW in controls, the difference was 0.06 BW), most likely attributed to differences in self-selected walking speed between previous study (Lee, Chang & Jeon, 2020) and this current study (average 0.55 m/s in Lee, Chang & Jeon’s study *vs.* 0.17 m/s in the present study). Both studies demonstrated, despite these differences in walking speeds, that the non-paretic limb of stroke survivors stayed functionally different from the dominant limb of healthy controls. Lee, Chang & Jeon (2020) noticed increased lateral foot placement relative to the center of mass during paretic limb stance phases, thereby requiring the non-paretic leg to employ a stabilization mechanism. Further increased hamstring activity in the non-paretic leg during late stance was observed by electromyography experiments revealing neuromuscular adaptations to offset paretic limb propulsion impairments (Raja, Neptune & Kautz, 2012). These compensatory tendencies presumably originate from bilateral neuromotor deficits following a stroke, which diminish corticospinal drive and interhemispheric inhibition, hence impairing coordination even in the non-paretic limb (Lim & Madhavan, 2023). Also, prolonged dependency on the non-paretic limb may lead to secondary complications including disuse atrophy and unequal joint stress, hence preserving gait asymmetry (Alexander et al., 2009). Although the non-paretic limb keeps limited capacity for perturbation modulation (Haarman et al., 2017), its functional profile differed significantly from healthy limbs, marked by lower mechanical efficiency, greater metabolic costs, and neuromuscular strategies incompatible with normal gait mechanics (Raja, Neptune & Kautz, 2012; Lee, Chang & Jeon, 2020). These findings taken together highlighted the need of bilateral rehabilitation paradigms in order to address basic neuromotor and biomechanical anomalies in post-stroke gait recovery.

**Table 3:** Mean and variabilities of all dependent variables using walking speed a covariate. Cgrade0: healthy controls walked on the 0% grade incline, Cgrade6: healthy controls walked on the 6% grade incline, Pgrade0: stroke survivors walked on the 0% grade incline, and Pgrade6: stroke survivors walked on 6% grade incline. NS: not significant. Values: Means (Standard Deviation). Conds: 0 vs. 6 grade incline. Groups: Stroke vs. Healthy controls. Speeds: walking speed as a covariate. F1 and F3: the values of first and second peak of vertical ground reaction force during stance phase (unitless: N/body weight * g). F1V and F3V: the coefficient of variation of F1 and F3 values within 60 gait cycles (unitless). F2 the value at the valley point of vertical ground reaction force during stance phase (unitless: N/body weight * g). F2V: the coefficient of variation of F2 values within 60 gait cycles (unitless). TimetoF1, TimetoF2, and TimetoF3: the time to these points (unit: % of stance cycle). TimetoF1V, TimetoF2V, and TimetoF3V: the coefficient of variation of TimetoF1, TimetoF2, TimetoF3 values within 60 gait cycles (unitless). J1, J2, J3 and J4: the impulses (unit: (N/Body weight *g) * time interval). J1V, J2V, J3V, and J4V: the coefficient of variation of J1, J2, J3 and J4 values within 60 gait cycles (unitless).

	Cgrade0	Cgrade6	PGrade0	PGrade6	Conds	Groups	Speed	Conds x groups	Conds x speed
F1	1.09 (0.09)	1.14 (0.09)	1.03 (0.04)	1.05 (0.05)	*F* = 4.502 *p* = 0.044	*F* = 4.957 *p* = 0.035	*F* = 32.719 *p* < 0.001	NS	NS
F1V	2.28 (0.51)	2.74 (0.44)	1.80 (0.36)	2.48 (0.79)	*F* = 8.656 *p* = 0.007	NS	NS	NS	NS
F2	0.95 (0.09)	0.89 (0.11)	0.99 (0.05)	0.96 (0.06)	*F* = 8.972 *p* = 0.006	*F* = 19.081 *p* < 0.001	*F* = 13.099 *p* = 0.001	NS	NS
F2V	2.22 (0.29)	2.71 (0.42)	1.81 (0.36)	2.19 (0.69)	*F* = 7.133 *p* = 0.013	*F* = 5.687 *p* = 0.025	NS	NS	NS
F3	1.05 (0.07)	1.12 (0.04)	1.05 (0.04)	1.01 (0.05)	NS	NS	NS	*F* = 10.677 *p* = 0.003	NS
F3V	2.31 (0.23)	2.72 (0.32)	1.75 (0.53)	2.11 (0.62)	*F* = 6.115 *p* = 0.021	*F* = 6.237 *p* = 0.019	NS	NS	NS
TimetoF1	29.86 (3.83)	28.31 (3.72)	33.44 (5.53)	33.97 (6.07)	NS	*F* = 8.631 *p* = 0.007	NS	NS	NS
TimetoF1V	6.71 (0.78)	8.89 (1.13)	7.47 (0.53)	10.82 (2.03)	NS	*F* = 15.997 *p* < 0.001	NS	NS	NS
TimetoF2	50.35 (2.52)	47.84 (3.36)	48.4 (4.71)	45.91 (5.44)	*F* = 7.291 *p* = 0.013	NS	NS	NS	NS
TimetoF2V	4.14 (0.97)	5.11 (0.71)	7.35 (0.93)	8.41 (1.47)	*F* = 4.503 *p* = 0.044	*F* = 21.973 *p* < 0.001	NS	NS	NS
TimetoF3	69.64 (2.48)	71.45 (2.35)	64.36 (6.34)	62.07 (5.16)	NS	*F* = 7.007 *p* = 0.014	NS	*F* = 6.599 *p* = 0.017	NS
TimetoF3V	2.24 (0.45)	2.73 (0.36)	4.02 (0.79)	5.11 (0.79)	*F* = 6.434 *p* = 0.018	*F* = 33.543 *p* < 0.001	NS	NS	NS
J1	22.99 (4.22)	25.08 (3.78)	19.12 (2.95)	21.18 (2.94)	*F* = 3.94 *p* = 0.05	*F* = 4.828 *p* = 0.037	*F* = 28.56 *p* < 0.001	NS	NS
J1V	5.55 (1.16)	7.87 (1.42)	9.39 (2.94)	11.45 (3.72)	NS	*F* = 5.14 *p* = 0.032	NS	NS	NS
J2	20.04 (3.33)	21.80 (3.92)	13.06 (2.49)	14.77 (2.61)	*F* = 10.927 *p* = 0.003	*F* = 7.605 *p* = 0.011	NS	NS	NS
J2V	7.30 (0.87)	9.35 (1.02)	10.34 (2.41)	12.69 (2.00)	NS	*F* = 23.67 *p* < 0.001	NS	NS	NS
J3	20.59 (4.45)	24.16 (4.14)	15.98 (3.22)	15.47 (4.20)	NS	*F* = 4.8 *p* = 0.038	NS	NS	NS
J3V	9.11 (1.01)	11.44 (1.63)	15.23 (3.29)	15.11 (4.17)	NS	*F* = 10.33 *p* = 0.004	NS	NS	NS
J4	21.91 (2.42)	21.14 (1.89)	17.89 (2.02)	19.71 (2.25)	NS	*F* = 14.982 *p* = 0.001	NS	*F* = 5.82 *p* = 0.023	NS
J4V	7.45 (0.69)	7.88 (0.97)	10.44 (1.82)	11.32 (0.95)	NS	*F* = 25.47 *p* < 0.001	NS	NS	NS

The current work showed significantly higher F2 magnitude during mid-stance of vertical GRFs in stroke survivors than in healthy Controls. Whether shallower or abnormally deep, deviations in the F2 revealed underlying gait problems, and the F2 suggested important biomechanical and neuromuscular control mechanisms during stride. In general, healthy people had F2 between 80% and 90% of BW (Winter, 2009). While patients with knee osteoarthritis showed F2 values matching with a crouched gait pattern (90–100% BW, (Costello et al., 2021), ataxic gait profiles were linked with much reduced F2 magnitudes (60–70% BW, Martino et al., 2014). A shallower or nonexistent mid-stance valley in the vertical GRF profile is characteristic of compensatory gait strategies or pathological conditions. Usually, this valley corresponds to a period of reduced vertical GRF as the body’s center of gravity rises above the stance limb, thereby facilitating effective energy transfer and dynamic balance control (Winter, 2009). Diminished or absent valleys suggest an adaptive minimization of vertical displacement to enhance stability or reduce discomfort (Costello et al., 2021). Such patterns are common in populations with joint pain, osteoarthritis, or post-injury adaptations, where flatter GRF waveforms, characterized by lower peaks and elevated mid-stance forces, reflected stiffer gait strategies (Evans-Pickett et al., 2020; Costello et al., 2021). For instance, reduced peak amplitudes and consistent mid-stance forces observed in those with knee osteoarthritis or those within 12 months of anterior cruciate ligament replacement suggest the presence of compensatory kinematic changes (Evans-Pickett et al., 2020; Costello et al., 2021). Similarly, adopting a body weight support or assistive device can reduce valley magnitude and flatten the GRF curve (Barela et al., 2014).

The changed F2 profiles were linked not only with more musculoskeletal strain and less functional mobility but also with higher energy expenditure in the present group of stroke survivors (Nadeau, Gravel & Olney, 2001; Lauzière et al., 2014). Since the mid-stance valley has been defined as a fundamental biomechanical marker of aberrant gait (Evans-Pickett et al., 2020; Costello et al., 2021), our results highlighted the need of focused therapeutic approaches to solve compensatory mechanisms and restore normal force distribution patterns.

### Walking on 6% grade incline had a different impact on F3 and J3 between stroke survivors and healthy controls

This work focused especially on how stroke affected vertical GRFs during walking on a 6% grade incline, an underexplored terrain in post-stroke gait analysis. Key results showed that when moving from level (0%) to inclined (6%) walking, both stroke survivors and healthy controls showed higher F1 and lower F2. Interestingly, a different response showed inF3 and J3: stroke survivors showed lower F3 magnitudes and J3 amplitudes on the incline, whereas healthy controls showed higher F3 and J3 values under identical conditions. The noted decrease in F3 and J3 among stroke survivors could be a result of compromised paretic limb push-off capacity or compensatory techniques in non-paretic leg meant to prioritize stability over propulsive force generation during incline walking. This J3 interval particularly corresponded to the transition from mid-stance to terminal stance and push-off. This phase was critical for forward propulsion and efficient gait mechanics. These deficiencies in push-off mechanics during incline walking could be mostly related to maladaptive neuromuscular strategies and impaired ankle plantar flexor function in both paretic and non-paretic leg. For instance, an electromyographic study showed that paretic limb medial gastrocnemius activation failed to increase proportionately with incline demands, whereas the non-paretic limb medial gastrocnemius showed increased activation for compensating the deficits of the paretic leg during fast walking condition (Mohammadi et al., 2016). This differential adaptation to speed *versus* incline demands reflected a basic disturbance in paretic plantar flexor recruitment, further reducing the activation of plantar flexors in the non-paretic leg during incline walking (Son & Rymer, 2020). Also, stroke survivors showed more tibialis anterior activation and less medial gastrocnemius’ recruitment during incline walking than in controls, thereby producing an antagonistic muscle imbalance that destabilizes ankle mechanics in both legs (Yoon, An & Oh, 2013). Hip and knee flexion were increased to compensate inadequate ankle propulsion, revealing this aberrant ankle muscle co-activation redirected the mechanical work proximally in both legs (Zhang et al., 2022). Such adaptations increased metabolic cost and failed to solve basic issues in plantar flexor force generation. Impaired sensory integration may exacerbate propulsion deficits: diminished proprioception from paretic plantar flexors reduced reflexive push-off contributions, forcing greater dependence on already-impaired volitional motor pathways. During incline walking, this sensory-motor decoupling was more pronounced, where propulsion required precise modulation of ankle stiffness and force output in both legs (Frisk et al., 2017). These components taken together has created a biomechanical “push-off paradox” in stroke gait: paretic neuromuscular constraints promote compensatory strategies that prolong propulsion deficits while incline walking decreases plantar flexor output in paretic leg and non-paretic leg. Targeted interventions combining incline training with real-time biofeedback and plantar flexor Neuromuscular Electrical Stimulation could help recalibrate activation patterns (Chen et al., 2024), thereby addressing both mechanical and neurological causes of push-off impairments.

### The effect of stroke on temporal parameters of vertical GRF

During walking on a 0% grade incline (level walking), there were unexpectedly no statistically significant differences in the time to F1, time to F2, and time to F3 between the non-paretic legs of the stroke survivors and the dominant leg of the healthy controls. We proposed that neuromotor control prioritized amplitudes of GRFs over temporal parameters of GRFs, potentially elucidating these results. Our hypothesis could be support by several evidence. When individuals face environmental challenges (*e.g.*, curbs, uneven surfaces), spatial parameters, such as step length and foot placement, were more important for safe navigation than temporal parameters. When approaching challenges, stroke patients often slow down and take shorter steps, suggesting a compensatory prioritizing of spatial control for safety (Handelzalts et al., 2021). Exactly spatial control is absolutely essential to prevent tripping and guarantee sufficient foot clearance (Handelzalts et al., 2021). During a 6% grade incline walking, stroke patients spent less time in single-limb support on the paretic side, resulting in asymmetrical stance and swing lengths (Pan et al., 2024). This temporal asymmetry, a characteristic of post-stroke gait, was linked with poor motor control and balance and hence increased time to F1 and reduced time to F3 in the non-paretic leg during incline walking.

### Loading and unloading rate between stroke survivors and healthy controls

Our findings demonstrated elevated loading rates in both stroke survivors and healthy controls during walking on a 6% graded incline. However, stroke survivors exhibited a reduction in unloading rate, whereas healthy controls demonstrated an increased unloading rate. The loading/unloading rate of vertical GRF during incline walking was influenced by biomechanical adaptations to slope demands. While direct studies on incline walking were limited, the alternation in loading/unloading rate might be extrapolated from incline running mechanics and general gait mechanics. Compared to level running, incline running has demonstrated a lower peak vertical GRF and a reduced loading rate. For instance, the loading rate dropped by ∼40% at an 8% incline compared to level running; this change could be ascribed to changed foot strike patterns (*e.g.*, shorter steps, lower braking forces) and redistributed muscle activation to adapt slope demands. Incline walking also caused the body’s center of mass to move anteriorly, thereby stressing knee extensors and ankle plantar flexors. This lowered the impact peak (F1) and distributed forces over a longer stance duration, thereby lowering loading rate. These biomechanical adaptations matched the observed increased F1 in the current study, which helped to explain higher loading rates than in level walking. In the current study, during incline walking, an increase in unloading rate in healthy controls but a drop in unloading rate in stroke survivors was noted. The decreased unloading rate in stroke survivors may result from prolongation of the unloading phase compared to level walking. Such adaptation was result from the compensatory gait adjustments for increased propulsive demands on inclines, driven by paretic limb muscle weakness and/or sensory impairments in stroke survivors (Louie & Eng, 2016).

### Optimal variability

Variability has often been regarded as an error in gait patterns. High gait variability has been considered as a marker of poor dynamic balance and unsteady walking (Patel et al., 2021; Patel et al., 2022). Reducing gait variability has therefore become a major focus of post-stroke rehabilitation. Force-control training and other interventions targeted at enhancing motor control and force steadiness have shown to be more effective than strength training alone in lowering gait variability and hence promoting safer, more stable walking patterns (Patel et al., 2021). Contrary to expectations, in the present study, stroke survivors showed smaller variability than healthy controls in some variables such as F1, F2, and F3. These findings challenge conventional interpretations of gait variability and raise the possibility that healthy controls might present greater inconsistency in force application between consecutive stance phases compared to stroke survivors in certain conditions. These patterns of variability might reflect adaptive strategies used by stroke survivors to maintain balance during treadmill walking. Increasing evidence suggested that variability should not always be simply construed as an error (Harbourne & Stergiou, 2009); instead, it might represent a strategy to maintain a steady gait pattern across different environments. Specifically, it has been suggested that excessively low variability induces biomechanical rigidity and then limit adaptive capacity, which is a pattern characteristic of pathological or developmentally delayed states. On the other hand, excessively high variability introduces neuromuscular noise and instability, limiting adaptability and elevating error and injury risk. Both extremes, too little or too much variability, are associated with unhealthy, less adaptable movement patterns (Brach et al., 2005; Harbourne & Stergiou, 2009). Therefore, the decreased force variability in stroke survivors who had a stroke within three months compared to controls may be explained by the patients’ attempts to maintain consistency from one stance phase to another to ensure walking safety. The trade-off may manifest as an increase in temporal variability, a compensatory mechanism to maintain consistent amplitude of force peaks.

### The effect of speed as covariate

To our best knowledge, this study was the first to include walking speed as a covariate to investigate the differences in vertical GRF between subacute stroke survivors and matched controls. Surprisingly, we observed that the vertical GRF correlated with walking speed during the weight acceptance phase of gait but showed a non-existent correlation during the push-off phase in both groups. This observation may be attributed to the distinct mechanical roles and time constraints of each phase. The weight acceptance phase is the time from foot contact to mid-stance, during which the limb must rapidly decelerate the body’s center of mass and absorb the kinetic energy of the impact (Winter, 2009). This mechanical necessity for greater impact absorption and more rapid deceleration with reduced contact time creates a strong, direct correlation between speed and the magnitude of the first vertical GRF peak. Conversely, the push-off phase is the latter half of stance, dedicated to accelerating the center of mass forward and upward for the next step (Winter, 2009). The present observation suggested that the primary mechanism for increasing speed in the push-off phase was to change the direction and timing of the force application (making it more horizontal and more rapid), rather than simply increasing the peak vertical magnitude. Therefore, the second vertical peak’s magnitude increases with speed at a less direct rate than the first peak, leading to a weaker correlation.

## Limitations

A significant limitation of this study lied in its exclusive examination of the non-paretic leg in early post-stroke survivors, without a direct comparison to the paretic limb within the same participants. This emphasis provided insightful analysis of compensatory mechanisms, but it did not fully clarify the bilateral and inter-limb dynamics severely disturbed after stroke. Using cutting-edge analytical approaches, such cross-correlation analysis (Wang, Chien & He, 2025), could offer a more complete comparison of limb gait patterns. We acknowledge that the generalizability of these findings is limited by our sampling strategy. To mitigate the limited information provided previously, the Methods section has been updated to clarify that participants were recruited using convenience sampling from the inpatient and outpatient rehabilitation clinics of Ningbo Ninth Hospital, which may introduce a local bias. Furthermore, the inclusion criterion of a walking speed ≤0.8 m/s warrants reflection. This threshold (adapted from Perry et al., 1995) explicitly selects for individuals typically classified as limited community ambulators who exhibit persistent asymmetric gait patterns. This approach was intentionally designed to isolate the population most likely to rely on the compensatory mechanisms and altered force distribution patterns that were the focus of this study. Therefore, although this recruitment strategy introduces a potential selection bias, the findings in the present study are appropriately applied to this targeted subgroup of stroke survivors. To enhance generalizability, future research is necessary to validate these findings using a multi-site or population-based recruitment strategy and must include stroke survivors with walking speeds greater than 0.8 m/s to determine if the relationship between the altered GRF profiles and adverse functional outcomes is relevant across the entire spectrum of post-stroke community ambulation. A potential limitation concerns the current definition of the F1–F2–F3 (M-shape) characteristics, specifically the lack of a tolerance threshold for the F2 value. When the F2 value (valley) is very close in magnitude to the F1 and/or F3 peaks, it can resemble an inverse-U shape. In the present study, however, the time to F2 occurred approximately midway between F1 and F3 in these stroke survivors similar to healthy individuals, supporting the identification of the M-shape. Furthermore, to the best of our knowledge, no previous studies have established a tolerance threshold to distinguish M-shaped patterns from inverse-U GRF patterns in subacute stroke survivors. Therefore, future studies should develop a tolerance threshold to reliably differentiate between them, particularly in this stroke population. Another limitation was that only vertical GRF was measured due to the limitation of instrumented treadmill, and future studies needs to focus on investigating the GRFs in anterior-posterior and medial-lateral directions in stroke survivors. Finally, comorbid diseases or pathologies that may impact the outcome and comparability were not recorded in the present study. Future studies should include this information as these conditions may represent important confounders.

## Conclusions

This study provides new insights into the compensatory strategies employed by the non-paretic leg in early post-stroke survivors during treadmill walking, both on level ground and on a 6% incline. The results revealed different changes in vertical GRF profiles in the non-paretic leg compared to healthy controls, including lowered peak amplitudes, delayed timing to force peaks, and altered variability across several gait parameters. These findings suggest that certain patterns of variability may reflect adaptive strategies rather than simple motor errors. When negotiating challenges like inclines, early post-stroke gait adaptation appeared primarily dependent on spatial control (force amplitude and impulse) over temporal parameters. These biomechanical adaptations underline the complexity of post-stroke gait and emphasize the need for rehabilitation strategies that focus on enhancing motor control and force steadiness alongside conventional strength training to promote safer, more stable walking patterns. However, these results must be interpreted with caution given several key limitations. The generalizability of these findings is constrained by the sampling strategy, which relied on convenience sampling from a single hospital and introduced a local bias. Furthermore, the inclusion criterion of a walking speed ≤0.8 m/s restricted the sample to a targeted subgroup of limited community ambulators.

##  Supplemental Information

10.7717/peerj.20766/supp-1 Supplemental Information 1 Data

## Additional Information and Declarations

### Data Availability

The following information was supplied regarding data availability: The raw data is available in the Supplementary File.
